# Vaginal metabolic profiles during pregnancy: Changes between first and second trimester

**DOI:** 10.1371/journal.pone.0249925

**Published:** 2021-04-08

**Authors:** Luca Laghi, Sara Zagonari, Giulia Patuelli, Chenglin Zhu, Claudio Foschi, Sara Morselli, Maria Federica Pedna, Vittorio Sambri, Antonella Marangoni

**Affiliations:** 1 Department of Agro-Food Science and Technology, Centre of Foodomics, University of Bologna, Cesena, Italy; 2 Family Advisory Health Centres, Ravenna, Italy; 3 Microbiology, DIMES, University of Bologna, Bologna, Italy; 4 Unit of Microbiology, Greater Romagna Hub Laboratory, Pievesestina di Cesena, Italy; University of Illinois, UNITED STATES

## Abstract

During pregnancy, the vaginal microbiome plays an important role in both maternal and neonatal health outcomes. Throughout pregnancy, the vaginal microbial composition undergoes significant changes, including a decrease in overall diversity and enrichment with *Lactobacillus* spp. In turn, the modifications in the microbial profiles are associated with shifts in the composition of vaginal metabolites. In this study, we characterized the vaginal metabolic profiles throughout pregnancy at two different gestational ages, correlating them with a microscopic evaluation of the vaginal bacterial composition. A total of 67 Caucasian pregnant women presenting to the Family Advisory Health Centres of Ravenna (Italy) were enrolled and a vaginal swab was collected at gestational ages 9–13 weeks (first trimester) and 20–24 weeks (second trimester). The composition of the vaginal microbiome was assessed by Nugent score and women were divided in ‘H’ (normal lactobacilli-dominated microbiota), ‘I’ (intermediate microbiota), and ‘BV’ (bacterial vaginosis) groups. Starting from the cell-free supernatants of the vaginal swabs, a metabolomic analysis was performed by means of a ^1^H-NMR spectroscopy. From the first to the second trimester, a greater number of women showed a normal lactobacilli-dominated microbiota, with a reduction of cases of dysbiosis. These microbial shifts were associated with profound changes in the vaginal metabolic profiles. Over the weeks, a significant reduction in the levels of BV-associated metabolites (e.g. acetate, propionate, tyramine, methylamine, putrescine) was observed. At the same time, the vaginal metabolome was characterized by higher concentrations of lactate and of several amino acids (e.g. tryptophan, threonine, isoleucine, leucine), typically found in healthy vaginal conditions. Over time, the vaginal metabolome became less diverse and more homogeneous: in the second trimester, women with BV showed metabolic profiles more similar to the healthy/intermediate groups, compared to the first trimester. Our data could help unravel the role of vaginal metabolites in the pathophysiology of pregnancy.

## Introduction

In healthy reproductive-aged women, the vaginal microbiome is generally dominated by different species of *Lactobacillus* genus [1, 2]. Lactobacilli promote the maintenance of the vaginal homeostasis, preventing the colonization and growth of exogenous and endogenous adverse microorganisms [3–5]. Their protective role is exerted through various mechanisms, such as vaginal pH lowering, bioactive compounds production, competition for nutrients and adhesion sites, and modulation of host immune response [6, 7].

The depletion of lactobacilli, together with the increase of different species of anaerobic bacteria, can result in the switch from a normal vaginal environment to a polymicrobial dysbiosis known as bacterial vaginosis (BV) [8]. Molecular studies based on 16S rRNA gene have shown that BV is characterized by higher abundances of different anaerobes, including *Gardnerella vaginalis*, *Atopobium*, *Prevotella*, *Mobiluncus*, and *Veillonella* [9–10]. The marked changes in the bacterial communities are accompanied by profound alterations in the composition of vaginal metabolites: during BV, higher concentrations of various biogenic amines and short chain fatty acids (SCFAs), and low levels of some amino acids have been found in the vaginal fluids [11–12].

The composition of the vaginal microbiome can vary throughout a woman’s life in response to various factors, such as age, hormonal levels, pregnancy, pharmaceutical treatments, and urogenital infections.

In particular, during pregnancy, the vaginal microbiome undergoes important changes, including a significant decrease in overall diversity and richness, increased stability, and enrichment with *Lactobacillus* spp. [13–16].

Although many questions remain unsolved, it is well established that the vaginal microbiome plays a crucial role in both maternal and neonatal health outcomes [17–18]. Indeed, healthy pregnancies are characterized by a low bacterial diversity and an enrichment of *Lactobacillus*, whereas reduced lactobacilli and increased bacterial diversity are associated with several pregnancy-related complications and preterm births [19–21].

While many studies have investigated the composition of the vaginal microbiome during pregnancy, less information is available regarding the vaginal metabolome in the different periods of gestation [22–24]. Therefore, in this study we characterized and compared the vaginal metabolomic profiles throughout pregnancy at two different gestational ages (i.e., first and second trimester), correlating them with a microscopic evaluation of the vaginal bacterial composition and various clinical and demographic parameters.

## Materials and methods

### Study group and sample collection

From April 2019 all the Caucasian pregnant women presenting to the Family Advisory Health Centres of Ravenna (Italy) for prenatal care were enrolled.

Exclusion criteria were the following: (i) age < 18 years; (ii) HIV-positivity; (iii) body mass index (BMI) > 33; (iv) medically assisted procreation; (v) use of any antibiotics in the past month; (vi) use of vaginal douches or topical agents in the last two weeks; (vii) presence of uncontrolled chronic diseases (e.g., diabetes, autoimmune disorders, malignancies); (viii) drug addiction or heavy smokers (> 15 cigarettes/day). Moreover, women with urogenital infections due to sexually transmitted pathogens (i.e., *Chlamydia trachomatis*, *Neisseria gonorrhoeae*, *Trichomonas vaginalis*, *Mycoplasma genitalium*), aerobic vaginitis or symptomatic vulvo-vaginal candidiasis were excluded.

At gestational ages 9–13 weeks (first trimester) and 20–24 weeks (second trimester), women underwent a clinical visit. For all patients, demographic data and information about urogenital symptoms were recorded.

Two vaginal swabs were collected at each time point (first and second trimester). The first one (E-swab, Copan, Brescia, Italy) was used for microbiological diagnostic tests and Nugent score assessment. The second was collected with a sterile cotton bud, re-suspended in 1 ml of sterile saline, and stored at -80°C until use. Frozen vaginal swabs were thawed, vortexed for 1 min and removed from the liquid. The liquid was centrifuged at 10000 × *g* for 15 min, and cell-free supernatants were used for metabolomic analysis, as described below.

A written informed consent was obtained from all subjects and the study protocol was approved by the Ethics Committee of Romagna (CEROM) (n° 2032 of 21^st^ February 2018). This study was carried out in accordance with the Declaration of Helsinki, following the recommendations of the Ethics Committee.

### Microbiological investigations

Commercial nucleic acid amplification techniques (NAATs) were used for *C*. *trachomatis*, *N*. *gonorrhoeae*, *T*. *vaginalis* and *M*. *genitalium* detection (Seeplex STI Master Panel 1, Seegene). Microscopic examination and cultures were performed for candidiasis and aerobic vaginitis diagnosis [25, 26].

The microbial load of vaginal *Candida* spp. was estimated by a semi-quantitative culture.

The composition of the vaginal microbiome was assessed by a Gram stain scoring system (Nugent score), evaluating for the presence of different bacterial morphotypes (*Lactobacillus* spp., *Gardnerella vaginalis* and *Mobiluncus* spp.) [27]. Based on this score, women were divided into 3 groups: ‘H’ (score 0–3; normal lactobacilli-dominated microbiota), ‘I’ (score 4–6; intermediate microbiota), ‘BV’ (score 7–10; bacterial vaginosis) [28].

### Metabolomic analysis

Metabolomic analysis was performed by means of a ^1^H-NMR spectroscopy starting from 700 μl of the cell-free supernatants of the vaginal swabs, added to 100 μl of a D_2_O solution of 3-(trimethylsilyl)-propionic-2,2,3,3-d4 acid sodium salt (TSP) 10 mM set to pH 7.0.

^1^H-NMR spectra were recorded at 298 K with an AVANCE III spectrometer (Bruker, Milan, Italy) operating at a frequency of 600.13 MHz, equipped with Topspin software (Ver. 3.5) [29]. The signals originating from large molecules were suppressed by a CPMG filter of 400 echoes, generated by 180° pulses of 24 μs separated by 400 μs [30]. The signals were assigned by comparing their multiplicity and chemical shift with Chenomx software data bank (ver 8.3, Chenomx Inc., Edmonton, Alberta, Canada).

### Statistical analysis

Data were analysed with Prism 5.02 version for Windows (GraphPad Software, San Diego, CA, United States) and R computational language. Differences in metabolic profiles among experimental groups were assessed by a Wilcoxon matched-pairs signed rank test (first trimester vs second trimester) or by Kruskal-Wallis test followed by Dunn’s Multiple Comparison test (H vs I vs BV).

A two-way ANOVA followed by Tukey *post hoc* test was used to assess significant differences between the groups (H vs I vs BV), considering contemporary the two vaginal samples for each woman. A robust principal component analysis (rPCA) was built on the centred and scaled concentrations of the metabolites showing significant differences between these groups.

Metabolite concentrations were correlated to demographic and clinical data by calculating Spearman correlation coefficient. A *P*-value < 0.05 was considered as statistically significant.

## Results

### Study population

A total of 67 Caucasian pregnant women with a mean age of 31.3 ± 4.8 years (min-max: 21–44) were included in the study. At the time of enrolment, the mean BMI was 23.6 ± 3.3 (min-max 18.8–31.9).

Only 6 of the 67 women (8.9%) were smokers, with a frequency ranging from 4 to 10 cigarettes/day.

At the first trimester (gestational age: 10.3 ± 1.5 weeks), 33 (49.2%) women showed a normal lactobacilli-dominated vaginal flora (Nugent score: 0–3), 26 (38.8%) were characterized by an intermediate microbiota (Nugent score; 4–6), whereas the remaining 8 (12%) harboured a BV-associated microbial composition.

Conversely, at the second trimester of pregnancy (gestational age: 22.5 ± 0.8), a greater number of women showed a normal microbiota (45 women; 67.1%), with a reduction of cases of dysbiosis (23.8% intermediate microbiota; 8.9% BV-associated flora).

At the first trimester, all the women (including those with a BV-related microbiota) denied the presence of urogenital symptoms, whereas in the second trimester 4 women (3 of them with intermediate/BV microbiota) reported the presence of vaginal discharge.

A vaginal colonization by *Candida* spp. was found in 11 women (16.4%) both at the first and second trimester.

### Vaginal metabolome profiles over time

A total of 63 metabolites were detected and quantified by ^1^H-NMR spectroscopy. Molecules mainly belonged to the groups of SCFAs, organic acids, amino acids, and biogenic amines (S1 Table).

As shown in Table 1, a total of 33 vaginal metabolites showed significantly different concentrations going from the first to the second trimester of pregnancy. The major changes concerned the increase in the levels of lactate and of several amino acids (e.g., tryptophan, phenylalanine, threonine, serine, glycine, aspartate, glutamate, isoleucine, leucine), together with the depletion of glucose, organic acids (acetate, propionate) and biogenic amines (tyramine, methylamine, putrescine).

**Table 1:** Molecules whose concentration (mM, mean ± SD) showed significant differences between the first and the second trimester of pregnancy.

	First trimester (mM; mean ± SD)	Second trimester (mM; mean ± SD)	*P* value	Variation
Adenine	0.011 ± 0.007	0.014 ± 0.008	0.002	↑
Tryptophan	0.0091 ± 0.002	0.0099 ± 0.002	0.01	↑
Benzoate	0.0034 ± 0.001	0.0029 ± 0.0009	0.003	↓
Phenylalanine	0.027 ± 0.013	0.034 ± 0.012	< 0.0001	↑
Phenylpropionate	0.035 ± 0.015	0.042 ± 0.012	< 0.0001	↑
Tyramin	0.008 ± 0.01	0.005 ± 0.009	0.01	↓
Hydroxyphenylacetate	0.023 ± 0.01	0.026 ± 0.007	0.02	↑
Inosine	0.0048 ± 0.002	0.0042 ± 0.002	0.03	↓
UDP	0.013 ± 0.003	0.014 ± 0.003	0.002	↑
Uridine	0.009 ± 0.005	0.008 ± 0.004	0.001	↓
Maltose	0.20 ± 0.12	0.15 ± 0.11	< 0.0001	↓
Threonine	0.054 ± 0.018	0.062 ± 0.018	0.001	↑
Lactate	2.39 ± 0.87	2.63 ± 0.80	0.002	↑
Serine	0.067 ± 0.039	0.078 ± 0.030	0.04	↑
Glycine	0.071 ± 0.024	0.090 ± 0.023	< 0.0001	↑
Glucose	0.056 ± 0.051	0.044 ± 0.035	0.01	↓
sn-Glycero-3-phosphocholine	0.009 ± 0.004	0.008 ± 0.004	0.002	↓
Creatinine	0.017 ± 0.011	0.013 ± 0.009	0.005	↓
Creatine	0.024 ± 0.008	0.028 ± 0.009	0.0003	↑
Aspartate	0.023 ± 0.009	0.026 ± 0.008	0.001	↑
Methylamine	0.0024 ± 0.004	0.0011 ± 0.001	< 0.0001	↓
Glutamine	0.028 ± 0.01	0.034 ± 0.01	< 0.0001	↑
Glutamate	0.26 ± 0.10	0.33 ± 0.08	< 0.0001	↑
5-Aminopentanoate	0.025 ± 0.02	0.026 ± 0.02	< 0.0001	↑
Methionine	0.009 ± 0.005	0.013 ± 0.006	< 0.0001	↑
Acetate	0.61 ± 0.73	0.46 ± 0.66	0.01	↓
Putrescine	0.007 ± 0.01	0.005 ± 0.01	0.04	↓
3-Hydroxyisovalerate	0.0014 ± 0.0004	0.0016 ± 0.0005	0.003	↑
Ethanol	0.036 ± 0.031	0.025 ± 0.017	0.003	↓
Propionate	0.026 ± 0.04	0.023 ± 0.07	< 0.0001	↓
Isoleucine	0.021 ± 0.01	0.026 ± 0.01	< 0.0001	↑
Valine	0.029 ± 0.01	0.031 ± 0.01	0.03	↑
Leucine	0.099 ± 0.04	0.12 ± 0.04	< 0.0001	↑

Differences were searched by Wilcoxon matched-pairs signed rank test and a P value < 0.05 was considered as significant.

### Vaginal metabolome profiles stratified by the vaginal bacterial composition

Differences were searched in the metabolic profiles of women, startified by the composition of the vaginal microbiota. Both at first and second trimester, our data highlighted differences in the concentration of several molecules, mainly between H and BV groups and between I and BV groups (S2 and S3 Tables). Differences between the groups were more marked in the first (e.g. 35 molecules differed significantly between H and BV) compared to the second trimester (e.g. 18 molecules differed significantly between H and BV). Globally, in accordance with previous investigations [11, 12], BV women were characterized by higher levels of biogenic amines, organic acids (especially SCFAs) and alanine, while higher levels of phenylpropionate, and diverse aminoacids (e.g. tryptophan, phenyalanine, threonine, serine, isoleucine, leucine) were associated to the H and I status.

**Fig 1:**
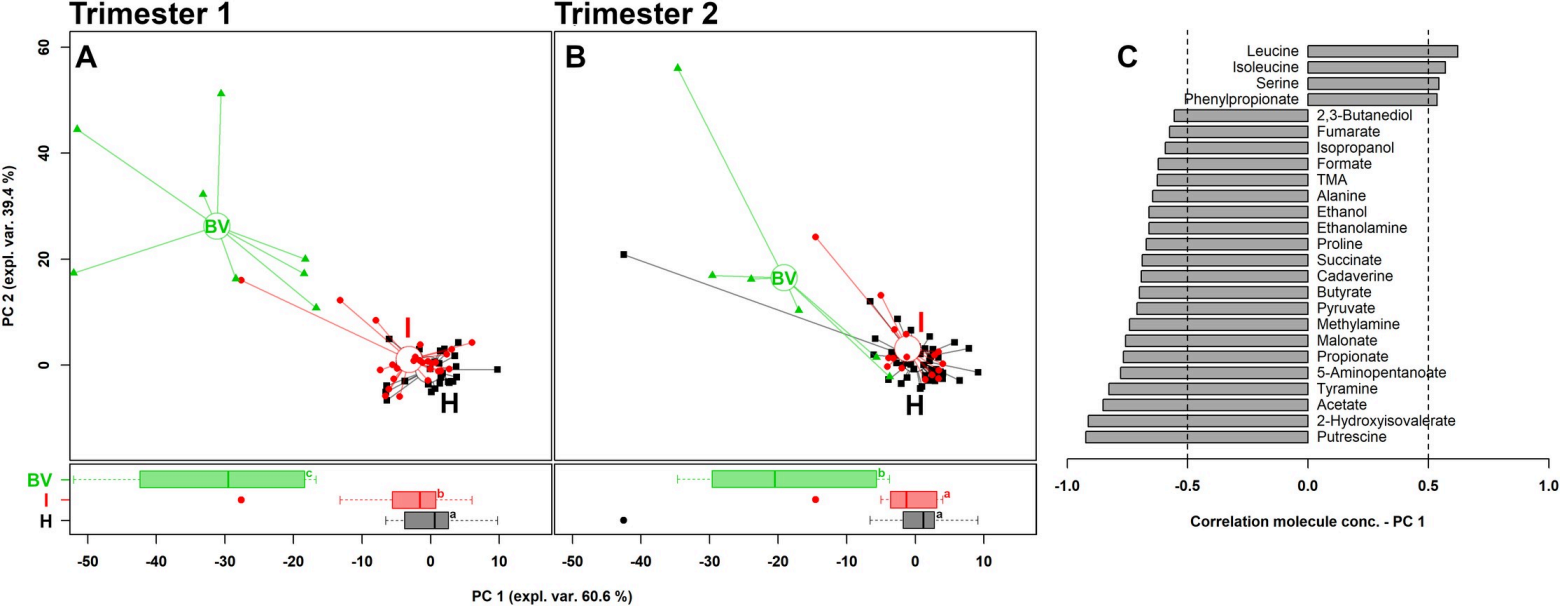
rPCA model built on the centred and scaled concentrations of the metabolites showing significant differences between groups. In the scoreplots (A: first trimester; B: second trimester), women with a healthy vaginal status (H), an intermediate flora (I) and a BV-related microbiota (BV) are represented in black, red and green respectively, with lines connecting each subject to the median of its group. Below, the respective boxplots represent the distribution of PC1 values of the women groups (a, b, c: equal letters indicate absence of statistical significance whereas different letters highlight a significant difference between the groups). In the barplot (C), describing the correlation between the concentration of each molecule and its importance over PC1, dark grey bars highlight statistically significant correlations (*P*<0.05).

To obtain overviews of the differences among groups, rPCA models were calculated on the basis of the molecules statistically different between the groups, irrespective of the time of sampling (first or second trimester). Fig 1A and 1B describe the comparison H vs I vs BV in the first and second trimester, respectively. In the first trimester, the score plots showed a clear separation between H/I and BV metabolomes along PC1. Nevertheless, a significant difference, even if less marked, was also noticed between H and I groups. Contrariwise, in the second trimester, no differences were observed between H and I women and the metabolic profiles of BV-positive subjects tended to become less diverse than H/I groups, compared to first trimester (S1 Fig).

As visualized in the correlation plot along PC1 (Fig 1C), higher levels of leucine, isoleucine, serine and phenylpropionate characterized H and I groups, whereas higher concentrations of putrescine, 2-hydroxyisovalerate, acetate, tyramine, 5-aminopentanoate, propionate, malonate, methylamine, pyruvate, butyrate, cadaverine, succinate, proline and ethanolamine seemed to be the most significant fingerprints of BV.

### Correlations between vaginal metabolic profiles and demographic/clinical factors

Both at first and second trimester of pregnancy, we found a positive correlation between creatinine levels and BMI (first trimester: Spearman coefficient = 0.29, *P* = 0.01; second trimester: 0.37, *P* = 0.001), between glucose concentrations and age (first trimester: 0.33, *P* = 0.006; second trimester: 0.25, *P* = 0.03) and between choline levels and microbial loads of vaginal *Candida* spp. (first and second trimester: 0.30, *P* = 0.01). Moreover, at the first trimester, glucose levels were positively correlated with *Candida* loads (0.37, *P* = 0.001), whereas, in the second trimester, lactate (-0.36, *P* = 0.002) and 4-aminobutyrate (-0.34, *P* = 0.004) concentrations were negatively correlated with age. Finally, at the second trimester, we found a positive correlation between uracil (0.28; *P* = 0.02), uridine (0.26; *P* = 0.02) and O-acetylcholine (0.30; *P* = 0.01) and *Candida* vaginal loads.

Higher levels of acetate (*P* = 0.02) were found in the vaginal fluids of smorkers (mean ± SD: 0.89 ± 0.59 mM) compared to non smokers (mean ± SD: 0.58 ± 0.74 mM).

## Discussion

In this work, a high-throughput technique, i.e., ^1^H-NMR spectroscopy [31], was applied to unravel the vaginal metabolome fingerprint during the first two trimesters of pregnancy. For this purpose, 67 Caucasian pregnant women were studied at 9–13 and 20–24 weeks of gestational age, excluding subjects with conditions able to perturb per se the vaginal environment (e.g., STIs, antimicrobial compounds, chronic diseases). Vaginal metabolic profiles were analysed over time and stratifying women by the composition of the vaginal microbiota (i.e., Nugent score).

As previously reported [32–34] and in line with our findings, throughout pregnancy, the vaginal microbiota composition becomes less diverse and rich, being mainly dominated by *Lactobacillus* spp.

The shifts towards a less complex and more stable microbiota were clearly associated with profound changes in the vaginal metabolic profiles. Globally, over the weeks, a significant reduction in the levels of dysbiosis-associated metabolites (e.g. tyramine, methylamine, putrescine, acetate, propionate) was observed. At the same time, the vaginal metabolome was characterized by higher concentrations of lactate, 4-hydroxyphenylacetate, and diverse aminoacids (e.g. leucine, isoleucine, valine and serine), typically found in healthy vaginal conditions. In addition, a significant reduction in the levels of vaginal glucose and maltose was noticed, moving from the first to the second trimester of pregnancy.

These metabolic changes reflect the trend towards the increase in the relative abundance of lactobacilli. These microbes consume glucose and maltose, with the consequent production of higher levels of lactate [8–11]. Moreover, lactic acid bacteria are known producers of branched-chain amino acids [35], being the higher concentrations of some of them, such as valine, leucine, and isoleucine, another hallmark of the prevalence of lactobacilli in healthy women.

In parallel, the reduction in the presence of diverse anaerobic bacteria lead to lower levels of SCFAs (e.g. propionate and acetate) and biogenic amines [8–11].

In addition, over time, the vaginal metabolic composition became less diverse and more homogeneous: indeed, in the second trimester, women with BV showed metabolic profiles more similar to the healthy/intermediate groups, compared to the first trimester.

We can speculate that, in the second trimester, BV-affected women were characterized by a less-complex (or less-diverse) microbial composition with a reduced impact on vaginal ecosystem metabolism.

Further studies, including an in-depth evaluation of the vaginal microbiome (i.e., by means of 16s rRNA sequencing), are needed to better understand the dynamics that take place in the vaginal environment and to better relate vaginal metabolic profiles with peculiar microbial fingerprints.

The importance of deciphering the vaginal metabolic profiles lies in the fact that the composition of vaginal metabolome can significantly impact on maternal-foetal health [36]. It has been shown that specific vaginal molecules (e.g., acetone, ethylene glycol, formate, glycolate, isopropanol, methanol, and TMAO) can predict the risk of preterm birth, with a negative correlation with gestational age at birth and cervical length [24, 37].

Other interesting data emerged when metabolic profiles were correlated with demographic/clinical parameters.

At first, we found a correlation between vaginal *Candida* loads and higher concentrations of glucose, choline, O-acetylcholine, uracil, and uridine. Vulvo-vaginal candidiasis has been previously associated with an enrichment in vaginal levels of TMAO, taurine, methanol, isopropanol, O-acetylcholine, and glucose [10].

In this context, it is worth mentioning that high levels of glucose enhance the nutritive substrate of *Candida*, and increase fungal adhesion, by promoting the expression of binding molecules in vaginal epithelial cells [38].

Moreover, in our dataset, lactate and 4-aminobutyrate concentrations negatively correlated with the age of women, creatinine levels positively correlated with BMI, whereas higher levels of acetate were related to smoking behavior. An alteration in acetate level has not been reported by Nelson et al. [39], when the vaginal metabolome of smokers was evaluated in comparison with non-smokers. At the same time, we did not observe other alterations in metabolite abundances previously shown by these autors [39]. This could be due to the exclusion of the heavy smokers (>15 cigarettes/day) from our study, in combination with a progressive self-limitation of the number of cigarettes per day due to pregnancy.

It is well known that various factors (e.g., age, race, diet, sexual activity, smoking) can modify the vaginal microbial composition [39, 40]. Age and BMI can impact on the levels of circulating estrogens, changing the relative abundance of lactobacilli and other microbial taxa. In turn, microbial variations can lead to alterations of vaginal metabolic profiles.

We are fully aware of some limitations of the study: (i) the lack of important variables, affecting the vaginal environment in terms of microbial composition and produced metabolites (e.g., hormonal levels); (ii) the use of a microscopic score for vaginal microbiome assessment instead of in-depth molecular techniques (e.g., 16s rRNA sequencing).

In conclusion, we shed light on the vaginal metabolome composition during the first two trimesters of pregnancy: over time, the vaginal metabolic composition becomes less diverse and more homogeneous, enriched in several molecules usually found in healthy lactobacilli-dominated vaginal environments.

Understanding the vaginal metabolome may hold promise for unravelling the pathogenesis of preterm birth and may provide novel biomarkers to identify subjects at risk of maternal-foetal complications [41, 42]. Further investigations with a prospective longitudinal study design, sampling women at the third trimester and puerperium, would afford understanding the role of vaginal metabolome in the pathophysiology of pregnancy.

## Supporting information

S1 Fig Distances between H and BV groups along PC1 in the first and second trimester. (TIF)

S1 Table List of vaginal molecules detected and quantified by ^1^H-NMR spectroscopy. (DOCX)

S2 Table Concentration (mM) of vaginal metabolites determined by ^1^H-NMR in the first trimester of pregnancy. Results are expressed as mean ± standard deviation. H: healthy, BV: bacterial vaginosis, I: intermediate flora. Arrows indicate significant variations (*P* < 0.05) in metabolite concentration (↑ increase, ↓ decrease) between groups. Differences were searched by Kruskal-Wallis test followed by Dunn’s Multiple Comparison test.
(DOCX)

S3 Table Concentration (mM) of vaginal metabolites determined by ^1^H-NMR in the second trimester of pregnancy. Results are expressed as mean ± standard deviation. H: healthy, BV: bacterial vaginosis, I: intermediate flora. Arrows indicate significant variations (*P* < 0.05) in metabolite concentration (↑ increase, ↓ decrease) between groups. Differences were searched by Kruskal-Wallis test followed by Dunn’s Multiple Comparison test.
(DOCX)
