# Supplementary figures and images for: Vaginal metabolic profiles during pregnancy: Changes between first and second trimester
Source: PLoS One. 2021 Apr 8;16(4):e0249925. doi: 10.1371/journal.pone.0249925 (PMC8031435; doi:10.1371/journal.pone.0249925)

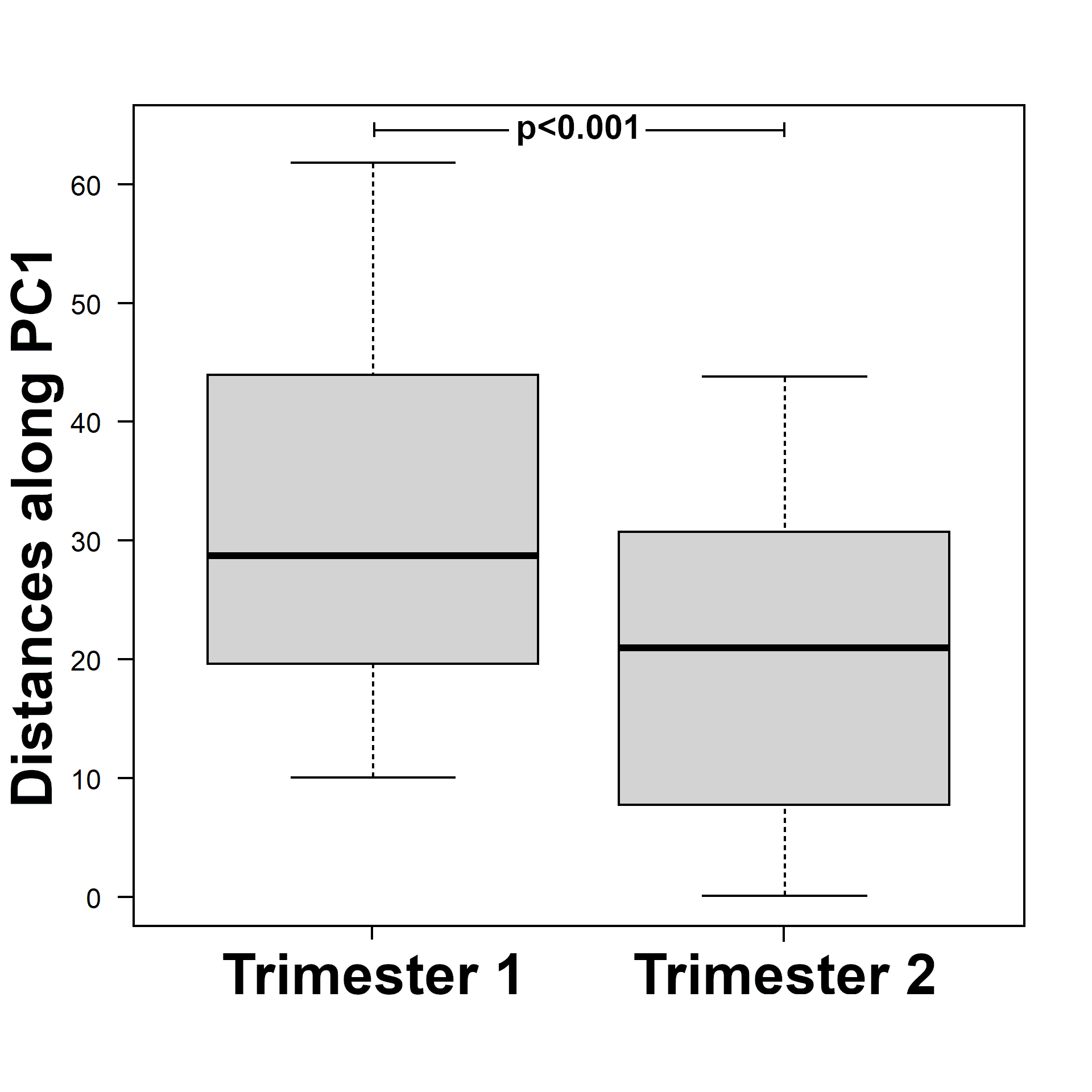

Supplement: S1 Fig — (TIF) [file pone.0249925.s001.tif]
