# Supplementary material for: Vaginal metabolic profiles during pregnancy: Changes between first and second trimester
Source: PLoS One. 2021 Apr 8;16(4):e0249925. doi: 10.1371/journal.pone.0249925 (PMC8031435; doi:10.1371/journal.pone.0249925)
Supplement: S1 Table — (DOCX) [file pone.0249925.s002.docx]

| **Amines** | **Organic acids** | **Amino acids** | **Alcohols** | **Sugars** | **Others** |
| --- | --- | --- | --- | --- | --- |
| Tyramine | Formate | Tryptophan | Methanol | Maltose | Hypoxanthine |
| Ethanolamine | Benzoate | Phenylalanine | Ethanol | Glucose | Adenine |
| Cadaverine | Phenylpropionate | Threonine | Isopropanol |  | Xanthine |
| TMA | 4-Hydroxyphenylacetate | Serine |  |  | Hippurate |
| DMA | 4-Hydroxyphenyllactate | Glycine |  |  | Inosine |
| Methylamine | Fumarate | Taurine |  |  | UDP |
| Putrescine | Ascorbate | Aspartate |  |  | Uridine |
|  | Lactate | Glutamate |  |  | Uracil |
|  | Malonate | Methionine |  |  | 1,3-Dihydroxyacetone |
|  | Succinate | Proline |  |  | Hydroxyacetone |
|  | Pyruvate | Alanine |  |  | sn-Glycero-3-phosphocholine |
|  | Acetate | Isoleucine |  |  | O-Acetylcholine |
|  | Butyrate | Valine |  |  | Choline |
|  | 3-Hydroxyisovalerate | Leucine |  |  | Creatinine |
|  | Propionate | Asparagine |  |  | Creatine |
|  | 2-Hydroxyisovalerate | Glutamine |  |  | Sarcosine |
|  |  |  |  |  | 4-Aminobutyrate |
|  |  |  |  |  | 5-Aminopentanoate |
|  |  |  |  |  | 2,3-Butanediol |

TMA=trimethylamine; DMA=dimethylamine

**S1 Table.**
