# Supplementary material for: Vaginal metabolic profiles during pregnancy: Changes between first and second trimester
Source: PLoS One. 2021 Apr 8;16(4):e0249925. doi: 10.1371/journal.pone.0249925 (PMC8031435; doi:10.1371/journal.pone.0249925)
Supplement: S2 Table — Results are expressed as mean ± standard deviation. H: healthy, BV: bacterial vaginosis, I: intermediate flora. Arrows indicate significant variations (P < 0.05) in metabolite concentration (↑ increase, ↓ decrease) between groups. Differences were searched by Kruskal-Wallis test followed by Dunn’s Multiple Comparison test. (DOCX) [file pone.0249925.s003.docx]

| **First trimester** | **H (n=33)** | **I (n=26)** | **BV (n=8)** | ***P* value** | **I vs H** | **BV vs H** | **BV vs I** |
| --- | --- | --- | --- | --- | --- | --- | --- |
| Formate | 0.04 ± 0.03 | 0.05 ± 0.05 | 0.14 ± 0.012 | 0.0008 |  | ↑ | ↑ |
| Adenine | 0.01 ± 0.006 | 0.01 ± 0.007 | 0.004 ± 0.001 | 0.0003 |  | ↓ | ↓ |
| Xanthine | 0.003 ± 0.001 | 0.004 ± 0.003 | 0.005 ± 0.004 | 0.01 | ↑ | ↑ |  |
| Tryptophan | 0.009 ± 0.002 | 0.009 ± 0.002 | 0.006 ± 0.002 | 0.007 |  | ↓ | ↓ |
| Phenyalanine | 0.02 ± 0.01 | 0.03 ± 0.01 | 0.01 ± 0.004 | 0.007 |  | ↓ | ↓ |
| Phenylpropionate | 0.03 ± 0.01 | 0.03 ± 0.01 | 0.01 ± 0.05 | 0.001 |  | ↓ | ↓ |
| Tyramine | 0.002 ± 0.004 | 0.002 ± 0.008 | 0.02 ± 0.05 | < 0.0001 |  | ↑ | ↑ |
| Fumarate | 0.0009 ± 0.0003 | 0.001 ± 0.0005 | 0.002 ± 0.0006 | 0.0004 |  | ↑ |  |
| Uracil | 0.005 ± 0.001 | 0.007 ± 0.002 | 0.006 ± 0.001 | 0.005 | ↑ |  |  |
| Threonine | 0.056 ± 0.018 | 0.056 ± 0.016 | 0.037 ± 0.015 | 0.02 |  | ↓ | ↓ |
| Serine | 0.076 ± 0.042 | 0.069 ± 0.031 | 0.026 ± 0.014 | 0.004 |  | ↓ | ↓ |
| Glucose | 0.03 ± 0.02 | 0.08 ± 0.07 | 0.06 ± 0.02 | 0.003 | ↑ |  |  |
| Taurine | 0.07 ± 0.02 | 0.09 ± 0.03 | 0.11 ± 0.04 | 0.0007 | ↑ | ↑ |  |
| O-acethylcholine | 0.0007 ± 0.0004 | 0.0009 ± 0.0005 | 0.0002 ± 0.0003 | 0.002 |  | ↓ | ↓ |
| Ethanolamine | 0.015 ± 0.004 | 0.016 ± 0.003 | 0.032 ± 0.01 | 0.0001 |  | ↑ | ↑ |
| Malonate | 0.001 ± 0.0005 | 0.001 ± 0.001 | 0.01 ± 0.006 | < 0.0001 |  | ↑ | ↑ |
| Creatinine | 0.016 ± 0.01 | 0.015 ± 0.01 | 0.026 ± 0.008 | 0.02 |  | ↑ | ↑ |
| Creatine | 0.022 ± 0.006 | 0.024 ± 0.007 | 0.033 ± 0.009 | 0.01 |  | ↑ | ↑ |
| Cadaverine | 0.007 ± 0.004 | 0.01 ± 0.008 | 0.03 ± 0.02 | 0.001 |  | ↑ | ↑ |
| TMA | 0.0006 ± 0.0002 | 0.0006 ± 0.0003 | 0.01 ± 0.01 | 0.0002 |  | ↑ | ↑ |
| DMA | 0.0009 ± 0.0003 | 0.0008 ± 0.0003 | 0.001 ± 0.0004 | 0.01 |  | ↑ | ↑ |
| Methylamine | 0.001 ± 0.0003 | 0.001 ± 0.001 | 0.01 ± 0.009 | 0.001 |  | ↑ | ↑ |
| Succinate | 0.05 ± 0.05 | 0.06 ± 0.1 | 0.46 ± 0.38 | 0.001 |  | ↑ | ↑ |
| Pyruvate | 0.03 ± 0.03 | 0.02 ± 0.03 | 0.13 ± 0.04 | 0.0002 |  | ↑ | ↑ |
| 5-Aminopentanoate | 0.01 ± 0.007 | 0.01 ± 0.009 | 0.08 ± 0.04 | < 0.0001 |  | ↑ | ↑ |
| Methionine | 0.009 ± 0.005 | 0.01 ± 0.005 | 0.005 ± 0.003 | 0.003 |  |  | ↓ |
| Proline | 0.004 ± 0.001 | 0.004 ± 0.004 | 0.01 ± 0.009 | < 0.0001 |  | ↑ | ↑ |
| Acetate | 0.36 ± 0.19 | 0.41 ± 0.39 | 2.29 ± 0.8 | < 0.0001 |  | ↑ | ↑ |
| Putrescine | 0.001 ± 0.0005 | 0.002 ± 0.008 | 0.05 ± 0.02 | < 0.0001 |  | ↑ | ↑ |
| Butyrate | 0.017 ± 0.01 | 0.018 ± 0.01 | 0.21 ± 0.3 | 0.009 |  | ↑ | ↑ |
| Alanine | 0.074 ± 0.02 | 0.075 ± 0.03 | 0.15 ± 0.04 | 0.0005 |  | ↑ | ↑ |
| Ethanol | 0.02 ± 0.008 | 0.03 ± 0.012 | 0.08 ± 0.07 | < 0.0001 |  | ↑ | ↑ |
| Isopropanol | 0.0009 ± 0.0004 | 0.001 ± 0.0007 | 0.002 ± 0.002 | 0.001 |  | ↑ | ↑ |
| 2,3-Butanediol | 0.002 ± 0.001 | 0.003 ± 0.002 | 0.006 ± 0.002 | 0.0008 |  | ↑ | ↑ |
| Propionate | 0.015 ± 0.01 | 0.017 ± 0.01 | 0.10 ± 0.07 | < 0.0001 |  | ↑ | ↑ |
| Isoleucine | 0.023 ± 0.01 | 0.023 ± 0.01 | 0.010 ± 0.007 | 0.006 |  | ↓ | ↓ |
| Leucine | 0.10 ± 0.04 | 0.10 ± 0.04 | 0.04 ± 0.02 | 0.004 |  | ↓ | ↓ |
| 2-Hydroxyisovalerate | 0.0004 ± 0.0004 | 0.0008 ± 0.001 | 0.008 ± 0.004 | < 0.0001 |  | ↑ | ↑ |

**S2 Table.**
