# Supplementary material for: Vaginal metabolic profiles during pregnancy: Changes between first and second trimester
Source: PLoS One. 2021 Apr 8;16(4):e0249925. doi: 10.1371/journal.pone.0249925 (PMC8031435; doi:10.1371/journal.pone.0249925)
Supplement: S3 Table — Results are expressed as mean ± standard deviation. H: healthy, BV: bacterial vaginosis, I: intermediate flora. Arrows indicate significant variations (P < 0.05) in metabolite concentration (↑ increase, ↓ decrease) between groups. Differences were searched by Kruskal-Wallis test followed by Dunn’s Multiple Comparison test. (DOCX) [file pone.0249925.s004.docx]

| **Second trimester** | **H (n=45)** | **I (n=16)** | **BV (n=6)** | ***P* value** | **I vs H** | **BV vs H** | **BV vs I** |
| --- | --- | --- | --- | --- | --- | --- | --- |
| Adenine | 0.013 ± 0.007 | 0.018 ± 0.008 | 0.005 ± 0.005 | 0.001 |  | ↓ | ↓ |
| Tryptophan | 0.01 ± 0.001 | 0.01 ± 0.002 | 0.006 ± 0.001 | 0.002 |  | ↓ | ↓ |
| Phenyalalanine | 0.036 ± 0.01 | 0.034 ± 0.01 | 0.023 ± 0.008 | 0.03 |  | ↓ |  |
| Phenylpropionate | 0.04 ± 0.01 | 0.04 ± 0.01 | 0.02 ± 0.01 | 0.01 |  | ↓ |  |
| Tyramine | 0.003 ± 0.006 | 0.006 ± 0.01 | 0.01 ± 0.01 | 0.01 |  | ↑ |  |
| Threonine | 0.060 ± 0.01 | 0.074 ± 0.02 | 0.044 ± 0.02 | 0.004 |  |  | ↓ |
| Lactate | 2.82 ± 0.76 | 2.26 ± 0.76 | 2.19 ± 0.70 | 0.01 | ↓ |  |  |
| O-acethylcholine | 0.0008 ± 0.0004 | 0.001 ± 0.0004 | 0.0004 ± 0.0004 | 0.04 |  |  | ↓ |
| Ethanolamine | 0.018 ± 0.005 | 0.018 ± 0.004 | 0.028 ± 0.01 | 0.02 |  | ↑ | ↑ |
| Creatine | 0.027 ± 0.009 | 0.028 ± 0.008 | 0.040 ± 0.01 | 0.01 |  | ↑ |  |
| Pyruvate | 0.023 ± 0.03 | 0.045 ± 0.04 | 0.10 ± 0.11 | 0.0002 | ↑ | ↑ |  |
| 5-Aminopentanoate | 0.021 ± 0.01 | 0.021 ± 0.007 | 0.078 ± 0.05 | 0.01 |  | ↑ |  |
| Proline | 0.005 ± 0.005 | 0.005 ± 0.002 | 0.01 ± 0.007 | 0.008 |  | ↑ |  |
| Acetate | 0.37 ± 0.58 | 0.30 ± 0.20 | 1.54 ± 1.02 | 0.01 |  | ↑ | ↑ |
| Putrescine | 0.002 ± 0.009 | 0.003 ± 0.008 | 0.03 ± 0.02 | 0.01 |  | ↑ |  |
| Alanine | 0.08 ± 0.02 | 0.10 ± 0.03 | 0.13 ± 0.04 | 0.001 | ↑ | ↑ |  |
| 3-Hydroxyisovalerate | 0.0016 ± 0.0005 | 0.0019 ± 0.0006 | 0.0012 ± 0.0003 | 0.03 |  |  | ↓ |
| 2,3-Butanediol | 0.003 ± 0.002 | 0.002 ± 0.002 | 0.005 ± 0.001 | 0.04 |  | ↑ |  |
| Propionate | 0.02 ± 0.08 | 0.008 ± 0.005 | 0.09 ± 0.07 | 0.03 |  | ↑ | ↑ |
| Isoleucine | 0.028 ± 0.009 | 0.029 ± 0.01 | 0.010 ± 0.003 | 0.0005 |  | ↓ | ↓ |
| Leucine | 0.12 ± 0.04 | 0.12 ± 0.04 | 0.05 ± 0.01 | 0.001 |  | ↓ | ↓ |
| 2-Hydroxyisovalerate | 0.0005 ± 0.0008 | 0.0004 ± 0.0004 | 0.006 ± 0.004 | 0.0006 |  | ↑ | ↑ |

**S3 Table.**
